# Complications and Outcomes of Primary Phacotrabeculectomy with Mitomycin C in a Multi-Ethnic Asian Population

**DOI:** 10.1371/journal.pone.0118852

**Published:** 2015-03-16

**Authors:** David Z. Chen, Victor Koh, Chelvin Sng, Maria C. Aquino, Paul Chew

**Affiliations:** 1 Department of Ophthalmology, National University Health System, Singapore, Singapore; 2 Yong Loo Lin School of Medicine, National University of Singapore, Singapore, Singapore; Bascom Palmer Eye Institute, University of Miami School of Medicine;, UNITED STATES

## Abstract

**Purpose:**

To determine the occurrence of intraoperative and postoperative complications up to three years after primary phacotrabeculectomy with intraoperative use of Mitomycin C (MMC) in primary open angle (POAG) and primary angle closure glaucoma (PACG) patients, and the effect of postoperative complications on surgical outcome.

**Methods:**

Retrospective review of 160 consecutive patients with POAG (*n* = 105) and PACG (*n* = 55), who underwent primary phacotrabeculectomy with MMC at the National University Hospital, Singapore, from January 1, 2008 to December 31, 2010. Data was collected using a standardized form that included patient demographic information, ocular characteristics and postoperative complications, including hypotony (defined as intraocular pressure < 6 mmHg), shallow anterior chamber (AC) and hyphema.

**Results:**

The mean age ± standard deviation (SD) of patients was 68.2 ± 8.2 years. No patient lost light perception during duration of follow-up. 77% of the postoperative complications occurred within the first month only. The commonest complications were hypotony (*n* = 41, 25.6%), hyphema (*n* = 16, 10.0%) and shallow AC (*n* = 16, 10.0%). Five patients (3.1%) required reoperation for their complications. Early hypotony (defined as hypotony < 30 days postoperatively) was an independent risk factor for surgical failure (hazard ratio [HR], 5.1; 95% CI, 1.6–16.2; p = 0.01). Hypotony with another complication was also a risk factor for surgical failure (p < 0.02).

**Conclusions:**

Hypotony, hyphema and shallow AC were the commonest postoperative complications in POAG and PACG patients after phacotrabeculectomy with MMC. Most complications were transient and self-limiting. Early hypotony within the first month was a significant risk factor for surgical failure.

## Introduction

Cataract and glaucoma are the top two causes of blindness in the world respectively and they frequently co-exist in the same eye [1]. In 2010, World Health Organization estimated that cataract is responsible for 51% of world blindness, representing about 20 million people (World Health Organization 2010), while another 4.5 million and 3.9 million people are blind due to open-angle and angle-closure glaucoma respectively [2]. In Singapore, about 1.14% and 0.5% of the population are affected by primary angle-closure glaucoma (PACG) and primary open angle glaucoma (POAG) respectively [3].

Treatment for patients with concurrent cataract and glaucoma includes sequential surgery and combined phacotrabeculectomy [4]. Combined phacotrabeculectomy has its advantages. First, it reduces risks of additional intra-ocular surgeries, because frequently after trabeculectomy, there is rapid progression of lens opacities into visually significant cataracts that require cataract surgery [5]. Secondly, sequential cataract surgery is associated with increased risk of trabeculectomy failure which may be minimised by combined phacotrabeulectomy [6]. Tan et al had previously determined that trabeculectomy with or without antimetabolites had low complications rates in Asian eyes at 1 year, with prolonged hypotony and bleb leak being the commonest complications [7]. However, longer-term data for combined phacotrabeculectomy with intraoperative Mitomycin C (MMC) is still lacking [8]. The effect of postoperative complications on phacotrabeculectomy success rates is also unclear. To address these gaps, we aimed to document the intraoperative and postoperative complications of combined phacotrabeculectomy with MMC in a multi-ethnic Asian population, and evaluate the impact of postoperative complications on surgical outcome.

## Materials and Methods

This was a retrospective review of 262 consecutive eyes of patients who had undergone primary phacotrabeculectomy with intraoperative MMC at National University Hospital (NUH) in Singapore between 1^st^ January 2008 and 31^st^ December 2010 (36-months period). The inclusion criteria for our study were as follows:

- Diagnosed with primary open angle glaucoma or primary angle closure glaucoma
- Diagnosed with visually-significant cataract on slit-lamp examination
- No previous surgical or laser operation on eye except laser peripheral iridotomy
- Intraocular pressure (IOP) measured with either Goldmann applanation tonometry or TonoPen
- Have ≥ 3 IOPs recorded before operation, measured on separate visits
- Completed at least one year of follow-up duration postoperatively

For those who underwent surgery for both eyes, only the first eye was included, consistent with the method used by similar previous studies [7, 9]. The study was conducted in accordance with the tenets of the World Medical Association’s Declaration of Helsinki and had ethics approval from the National Healthcare Group Domain Specific Review Board (NHG-DSRB), with waiver of informed consent. Patient information was anonymized and de-identified prior to analysis.

Glaucoma is defined as the presence of characteristic optic disc changes (thinning, excavation or focal notch of the neurosensory rim, or asymmetrical cupping between the eyes of > 0.2) and intraocular pressure (IOP) ≥ 21 mmHg on one or more visits preoperatively by Goldman applanation tonometry (GAT) with characteristic visual field changes compatible with glaucoma, defined as follows [10]:

1. Glaucoma Hemifield test outside normal limits, and
2. ≥ 3 contiguous points on the pattern deviation plot depressed at P < 5% level.

In addition, PACG was defined as glaucoma in which ≥ 180° of the posterior trabecular meshwork was not visible on non-indentation gonioscopy, with or without peripheral anterior synechiae [7].

### Data collection

Each medical record was reviewed and relevant data was transferred to a standardized form which included patient demographic information, type of glaucoma (POAG or PACG), relevant systemic conditions and intraoperative details. Preoperative and postoperative data were collected for best-corrected visual acuity (BCVA), IOP, as well as number and duration of glaucoma medication usage.

Postoperative complications were stratified into early (≤ 1 month), late (> 1 month), or recurrent (occurring more than once after surgery) [11]. A complication was considered severe if it required reoperation or resulted in a loss of BCVA by ≥ 2 lines on Snellen chart. Hypotony was defined as IOP < 6 mmHg on one or more occasions after surgery, and considered prolonged if it persisted for more than or equal to two weeks [11]. Shallow anterior chamber (AC) was defined as irido-corneal contact extending to within 1 mm of the pupil [11]. Hyphema was defined as microscopic or macroscopic blood in anterior chamber and/or iris. Bleb leak was defined as either a positive Siedel’s test or visible conjunctival leak from leak after surgery. All above definitions, as well as definitions for other less common complications, were also included in accordance with Guidelines on Design and Reporting of Glaucoma Surgical Trials [11].

### Surgical Procedures

All the patients underwent a standard operation performed by trained ophthalmologists, which comprised the following steps:

- Peri-bulbar or topical anaesthesia
- Fornix-based conjunctival flap in the superior nasal or temporal quadrant
- Creation of a 4 x 3 mm rectangular partial-thickness scleral flap with adequate hemostasis
- Sponges soaked in 0.4 mg/ml (0.4%) MMC inserted in the subconjunctival pocket for 5 minutes.
- Sponges removed and 0.4% MMC flushed out using 20 ml of balanced saline solution.
- Phacoemulsification of cataract and acrylic intraocular lens implantation
- Sclerostomy creation with straight beaver blade assisted by Kelly's punch
- Surgical peripheral iridectomy
- Scleral flap closure with 10-O nylon sutures until there was a slow egress of aqueous
- Conjunctival closure with 8-O vicryl sutures

After surgery, all patients were discharged with antibiotic and steroid eye drops from three-hourly to four times a day for the first week after surgery. Antibiotic eye drops were stopped one month from surgery date. Steroid eye drops were continued for up to six months with slow taper depending on the bleb morphology, IOP and AC depth as assessed by an experienced glaucoma consultant. In general, after an uncomplicated surgery, patients were reviewed at approximately one day, one week, one month, three months and every six months thereafter.

### Primary and Secondary Outcome Measures

The primary outcome measure was the cumulative occurrence of postoperative complications at 3-years for all patients after combined phacotrabeculectomy with MMC. All complications occurring after 3-years were censored. Secondary outcome measures included risk factors for the commonest postoperative complications, correlation between postoperative complications and failure, incidence of intraoperative complications, and number of reoperations required. Time to failure was defined as the time from surgical treatment to time to 1) loss of visual acuity to no light perception, or 2) first of two consecutive follow-up visits after one month in which the patient had persistent IOP < 6mmHg or greater than stipulated upper limit (15 mmHg, 18 mmHg and 21 mmHg), or 3) reoperation for glaucoma, whichever occurred first.

### Statistical Analysis

Statistical analysis was performed using SPSS 21.0 (SPSS Inc, Chicago, Illinois). For categorical variables, Pearson’s chi-square test or Fisher’s exact test was performed. For continuous variables, an independent t-test was performed for normally-distributed samples, while a corresponding non-parametric test was used for non-normally distributed samples. Multivariate logistics regression (including demographic information, preoperative IOP and type of glaucoma) was performed for the top three commonest complications. Kaplan Meier survival analysis was performed for the time to hypotony, hyphema, shallow AC, as well as time to failure. A p value of less than 0.05 was considered statistically significant.

## Results

### Baseline characteristics

A total of 262 total operations were performed between January 2008 and December 2010. Of these, 67 (25.6%) were excluded due to the following reasons: postoperative follow-up duration of less than one year (*n* = 32, 12.2%), preoperative IOP taken on less than three occasions (*n* = 16, 6.1%), previous intraocular operation/lasers except peripheral iridotomy performed such as laser trabeculoplasty, iridoplasty, retinal detachment surgery, pterygium surgery (*n* = 14, 5.3%), secondary glaucoma such as traumatic, neovascular, uveitic glaucoma (*n* = 5, 1.9%). 195 eyes of 168 patients fulfilled inclusion criteria. A total of 160 first operated eyes were included in the final analysis (eight eyes were excluded due to intra-operative phacoemulsification-related complications).

Baseline characteristics of patients were summarized in **Table 1**. PACG patients had significantly higher proportion of females (p = 0.01) and higher mean preoperative IOP (p < 0.001). Otherwise, the other demographic characteristics were similar between patients with POAG and PACG.

**Table 1:** Baseline characteristics of patients with primary open angle glaucoma and primary angle closure glaucoma.

Characteristics*	**All eyes (*n* = 160)**	**POAG eyes (*n* = 105)**	**PACG eyes (*n* = 55)**	**p-value^‡^**
**Patient demographic information**				
**Mean age, years (SD)**	68.2 (8.2)	68.2 (8.0)	68.1 (8.6)	0.93
**Gender (female), n (%)**	72 (45.0)	39 (37.1)	33 (60.0)	0.01
**Race, n (%)** ^§^				
**Chinese**	121 (75.6)	77 (73.3)	44 (80.0)	0.35
**Malay**	13 (8.1)	7 (6.7)	6 (10.9)	
**Indian**	13 (8.1)	10 (9.5)	3 (5.5)	
**Other Asians**	13 (8.1)	11 (10.5)	2 (3.6)	
**Diabetes mellitus**	60 (37.5)	43 (41.0)	17 (30.9)	0.21
**Ocular characteristics**				
**Left eye, n (%)**	73 (45.6)	48 (45.7)	25 (45.5)	0.98
**Preoperative IOP, mmHg (SD)**	17.2 (3.5)	16.4 (3.2)	18.6 (3.6)	< 0.001
**CDR (SD)**	0.65 (0.19)	0.66 (0.18)	0.64 (0.20)	0.42
**Pre-operative BCVA, median LogMAR (IQR)** ^**†**^	0.301 (0.176–0.398)	0.301 (0.176–0.398)	0.301 (0.176–0.398)	0.30
**Intra-ocular pressure lowering medications**				
**Number of medications, n (SD)**	1.6 (0.9)	1.6 (0.9)	1.7 (1.0)	0.55
**Mean duration of medication, months (SD)**	44.1 (38.4)	47.1 (40.4)	38.5 (33.9)	0.18

CDR = cup-disc ratio; IOP = intraocular pressure; IQR = interquartile range; LogMAR = logarithm of the minimum angle of resolution; PACG = primary angle closure glaucoma; POAG = primary open angle glaucoma; SD = standard deviation.

*Data presented as mean (standard deviation) or number (percentage), unless otherwise specified.

^†^Data presented as median (interquartile range).

^‡^p-values were calculated using chi-square test, independent t-test or Mann-Whitney U test as appropriate.

^§^p-value for race was calculated using Chinese and non-Chinese as categorical variables (chi-square test).

### Intraoperative complications

Ten patients (6.0%) experienced intraoperative complications. Of these, eight (4.8%) were phacoemulsification related (posterior capsular rupture [PCR]: *n* = 7, 4.2%; incomplete capsulorrhexis: *n* = 1, 0.6%), while two cases (1.2%) were trabeculectomy-related (conjunctival button hole: *n* = 1, 0.6%; full thickness scleral defect during scleral flap creation: *n* = 1, 0.6%). PCRs were treated intraoperatively with vitrectomy and sulcus placement of IOL, while an extracapsular cataract extraction was performed for the patient with incomplete capsulorrhexis.

### Postoperative complications

The mean follow-up duration ± standard deviation (SD) was 47 ± 14 months for all patients. No eye lost light perception during the duration of follow-up. 69 patients (43.1%) experienced at least one complication in the postoperative period. Of a total of 95 postoperative complications, most occurred within and resolved within the first month (*n* = 73, 76.8%). The top three commonest complications were hypotony (*n* = 41, 25.6%), hyphema and shallow AC (both *n* = 16, 10.0%). Of those with hypotony, the mean IOP was 13.1 ± 3.8 mmHg at 3 years postoperatively, as compared to a mean IOP of 14.9 ± 3.8 mmHg for those without hypotony (p = 0.049; number loss to follow up = 26, 16.3%). Seven patients (4.2%) experienced prolonged hypotony. A summary of postoperative complications was shown in **Table 2**. There was no significant difference in incidence of complications between POAG and PACG patients (POAG: *n* = 40, 38.1%; PACG: *n* = 29, 52.7%; p = 0.08). Six patients (3.8%) had severe complications that included shallow AC, hyphema, persistent inflammation more than three months, lens malposition, with five patients (3.1%) requiring reoperation. The patient with persistent inflammation also had worsening of BCVA by ≥ 2 Snellen lines. There was no significant difference in the incidence of severe complications between POAG and PACG patients (POAG: *n* = 2, 1.9%; PACG: *n* = 4, 7.3%; p = 0.18, Fisher’s Exact Test).

**Table 2:** Summary of all postoperative complications up to and including 3 years after surgery.

Complication*	**Early (*n* = 73)**	**Late (*n* = 13)**	**Early and late (*n* = 9)**	**Total (*n* = 95)**
**Hypotony**	31 (42.4)	5 (38.5)	5 (55.6)	41 (43.2)
**Hyphema**	16 (21.9)	0	0	16 (16.8)
**Shallow AC**	13 (17.8)	2 (15.4)	1 (11.1)	16 (16.8)
**Bleb leak**	5 (6.8))	0	0	5 (5.3)
**Choroidal detachment**	3 (4.1)	1 (7.7)	0	4 (4.2)
**Blebitis**	0	2 (15.4)	0	2 (2.1)
**Persistent inflammation**	0	0	2 (22.2)	2 (2.1)
**PVD/Macular hole**	2 (2.7)	0	0	2 (2.1)
**Aqueous misdirection syndrome**	1 (1.4)	0	0	1 (1.1)
**Dellen**	0	1 (7.7)	0	1 (1.1)
**Descemet membrane detachment**	0	1 (7.7)	0	1 (1.1)
**Lens malposition**	1 (1.4)	0	0	1 (1.1)
**Recurrent uveitis**	0	0	1 (11.1)	1 (1.1)
**Stitch granuloma**	0	1 (7.7)	0	1 (1.1)
**Tenon cyst**	1 (1.4)	0	0	1 (1.1)
**Endophthalmitis**	0	0	0	0

AC = anterior chamber; early = less than or equal to one month from operation; early and late = occurring at least once in each; late = more than one month from operation; PVD = posterior vitreous detachment.

*Data presented as number (percentage).

After adjusting for age, type of glaucoma (POAG versus PACG) and mean preoperative IOP, our results showed that male gender and non-Chinese race were independent risk factors for developing shallow AC after phacotrabeculectomy (male: odds ratio [OR] = 9.56, 95% confidence interval [95% CI] 1.84–49.67, p = 0.01; Chinese: OR 0.20, 95% CI 0.04–0.92, p = 0.04, **Table 3**). However, further subgroup analysis showed that neither race nor gender was a significant risk factor for developing shallow AC with concomitant hypotony (p ≥ 0.05 for both).

**Table 3:** Logistic regression analysis of the risk factors for hypotony, hyphema and shallow anterior chamber*.

	**Hypotony**		**Hyphema**		**Shallow AC**	
	**OR (95% CI)**	**p-value**	**OR (95% CI)**	**p-value**	**OR (95% CI)**	**p-value**
**Age**	1.03 (0.99, 1.08)	0.18	1.00 (0.94, 1.06)	0.92	0.98 (0.91, 1.04)	0.46
**Gender**						
**Male**	0.97 (0.46, 2.05)	0.93	3.20 (0.92, 11.2)	0.07	9.56 (1.84, 49.67)	0.01
**Female**	Reference		Reference		Reference	
**Race**						
**Chinese**	0.73 (0.21, 2.59)	0.73	1.40 (0.16, 11.99)	0.77	0.20 (0.04, 0.92)	0.04
**Malay**	0.58 (0.10, 3.51)	0.55	5.33 (0.43, 65.57)	0.19	0.17 (0.01, 2.20)	0.18
**Other Asians** ^**†**^	0.97 (0.18, 5.24)	0.97	1.34 (0.07, 25.28)	0.85	< 0.001	0.998
**Indians**	Reference		Reference		Reference	
**Type of glaucoma**						
**PACG**	1.49 (0.67, 3.32)	0.33	1.65 (0.50, 5.42)	0.41	2.76 (0.82 9.33)	0.10
**POAG**	Reference		Reference		Reference	
**Preop IOP**	1.03 (0.92, 1.15)	0.64	0.94 (0.79, 1.12)	0.48	1.11 (0.93, 1.33)	0.25

AC = anterior chamber; CI = confidence interval; IOP = intraocular pressure; OR = odds ratio; PACG = primary angle closure glaucoma; POAG = primary open angle glaucoma; preop = preoperative.

*Hypotony, hyphema and shallow AC were the top three commonest complications (in descending order).

^†^Other Asians excluding Chinese, Malays and Indians.

### Postoperative complications and success rates

**Fig 1:**
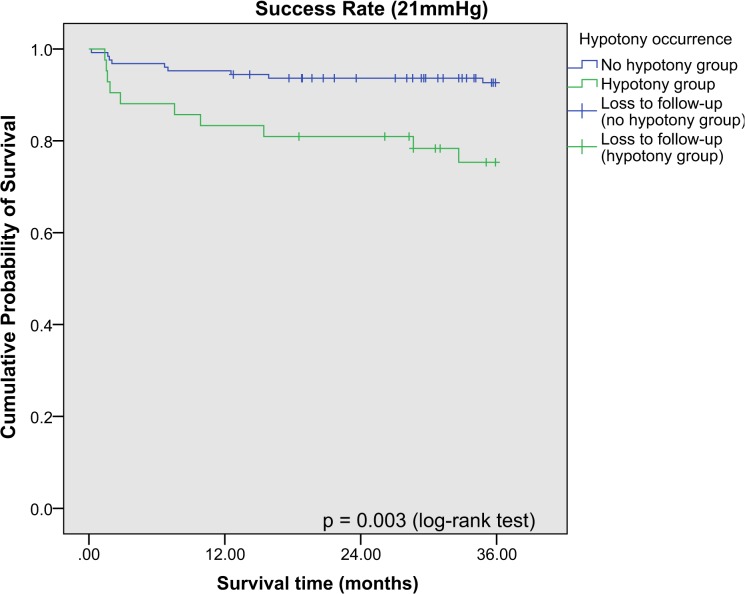
Kaplan Meier survival analysis of eyes with and without post-operative hypotony achieving surgical success (21mmHg). Failure was defined as loss of visual acuity to no light perception, or first of 2 consecutive follow-up visits after 1 month in which the patient had persistent intraocular pressure (IOP) < 6mmHg or > 21mmHg, or reoperation for glaucoma, whichever occurs first.

**Fig 2:**
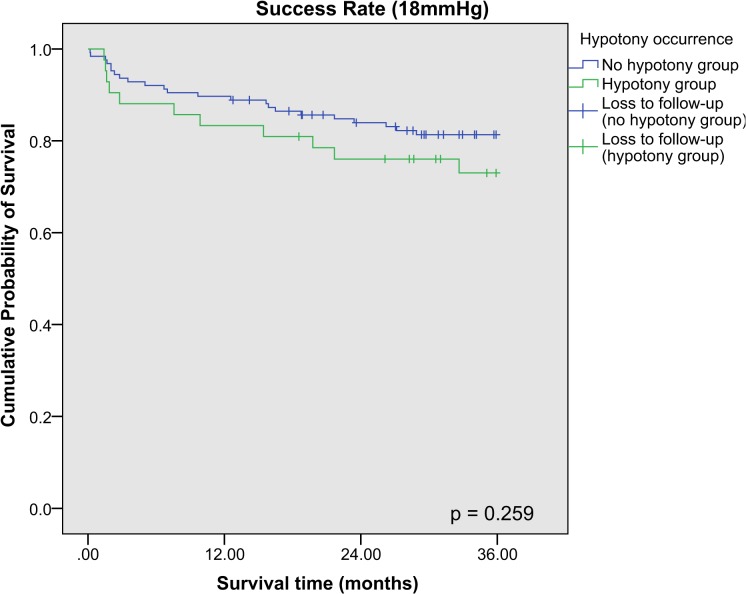
Kaplan Meier survival analysis of eyes with and without post-operative hypotony achieving surgical success (18mmHg). Failure was defined as loss of visual acuity to no light perception, or first of 2 consecutive follow-up visits after 1 month in which the patient had persistent intraocular pressure (IOP) < 6mmHg or > 18mmHg, or reoperation for glaucoma, whichever occurs first.

**Fig 3:**
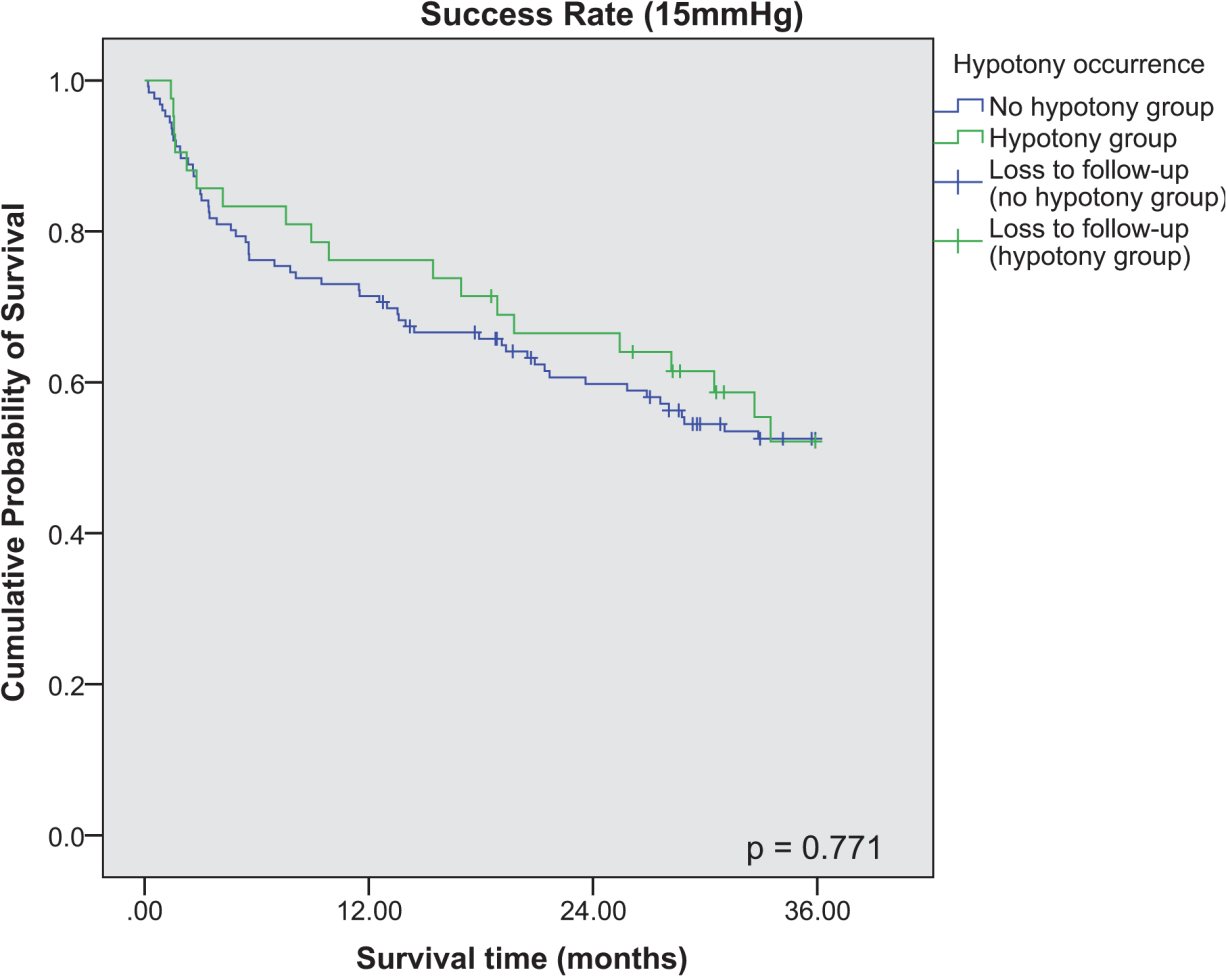
Kaplan Meier survival analysis of eyes with and without post-operative hypotony achieving surgical success (15mmHg). Failure was defined as loss of visual acuity to no light perception, or first of 2 consecutive follow-up visits after 1 month in which the patient had persistent intraocular pressure (IOP) < 6mmHg or > 15mmHg, or reoperation for glaucoma, whichever occurs first.

Kaplan-Meier survival analysis was performed for the success rates of combined phacotrabeculectomy at 21 mmHg, 18 mmHg and 15 mmHg respectively, and stratified according to the occurrence of hypotony postoperatively (**Fig. 1, Fig. 2, Fig. 3**). The 3-year cumulative incidence of failure (standard error [SE]) for 21 mmHg, 18 mmHg and 15 mmHg were 10.8% (2.5%), 18.5% (3.1%) and 45.9% (4.1%) respectively. Hypotony was associated with significantly higher risk of failure at 21 mmHg (cumulative percent (SE): with hypotony: 25.3% (7.0%); without hypotony: 5.9% (2.2%), p < 0.001), but not at 18 mmHg and 15 mmHg (p = 0.09 and p = 0.88 respectively). There was no difference between the cumulative incidence of failure (SE) between POAG and PACG eyes for 21 mmHg (cumulative percent (SE): POAG: 10.7% (0.03%); PACG: 11.1% (0.04%), p = 0.92), and for other definitions of failure (p > 0.05 for both). After adjusting for age, gender, race, preoperative IOP and type of glaucoma, our results showed that among factors influencing hypotony, hypotony with another simultaneous complication was an independent risk factor for failure at 21 mmHg, while both early and late hypotony were also significantly associated with failure (**Table 4**).

**Table 4:** Cox proportional hazard analysis for hypotony as a risk factor for surgical failure (Intraocular pressure > 21 mmHg).

Hypotony risk factors*	**Multivariable**	
	**HR (95% CI)**	**p-value**
**Onset of hypotony**		
**Early hypotony**	5.1 (1.6, 16.2)	0.01
**Late hypotony**	10.4 (1.6, 67.1)	0.01
**No hypotony**	Reference	
**Type of complications**		
**Hypotony only**	4.5 (1.2, 16.3)	0.02
**Hypotony with other complication(s)** ^**†**^	8.1 (2.4, 27.7)	0.001
**Other complications(s)**	Reference	

CI = confidence interval; HR = hazard ratio; early hypotony = hypotony occurring less than or equal to 30 days postoperatively; late hypotony = hypotony occurring later than 30 days postoperatively.

*Each model was adjusted independently for age, gender, race, type of glaucoma and mean preoperative intraocular pressures.

^†^Other complication(s) include: bleb leak, blebitis, choroidal detachment, choroidal effusion, descemet membrane detachment, hyphema, lens malposition, persistent inflammation shallow anterior chamber.

### Reoperations

18 patients (11.3%) required at least one surgical intervention postoperatively. The most commonly performed procedure was bleb needling with antimetabolite use (*n* = 7, 4.4%). Other trabeculectomy-related surgical interventions performed include glaucoma drainage device insertion (n = 2, 1.3%), AC reformation (*n* = 2, 1.3%), AC washout, bleb refashioning and irrigation and aspiration of hyphema (*n* = 1, 0.6% each). Two phacoemulsification-related interventions were performed: lens repositioning and soft lens matter removal (*n* = 1, 0.6% each). Other postoperative surgical procedures included the following: stitch granuloma removal, trans pars plana vitrectomy, laser peripheral iridotomy and conjunctival defect repair (*n* = 1, 0.6% each).

## Discussion

Combined phacotrabeculectomy with MMC is a commonly performed surgery on patients with concurrent cataract and glaucoma. In our study, the commonest postoperative complications up to three years were hypotony, hyphema and shallow AC respectively. There was a trend showing that PACG eyes had a higher complication rate as compared to POAG, although the difference was not statistically significant (p = 0.08). Most complications were self-limiting and did not necessitate further surgical intervention. The majority (77%) of postoperative complications occurred within the first month only, suggesting that the first postoperative month is the most important period for postoperative monitoring.

Similarly, the Trabeculectomy Outcomes Group Audit Study Group also emphasized the importance of intensive proactive postoperative care to reduce rates of surgical complication after trabeculectomy. In their audit of 428 eyes of 395 white patients with open-angle glaucoma, 183 out of 184 (99%) suture manipulations and 21 out of 22 (95%) of resuturings took place in the first three months, and they proceeded to recommend that close follow-up of patients and proactive intervention are necessary after trabeculectomy, though they did not specify strict duration [12].

Several other authors have also reported complication rates after trabeculectomy or phacotrabeculectomy [7, 9, 13]. A recent large retrospective case series of postoperative complications after phacotrabeculectomy in Asian eyes reported prolonged hypotony (defined as IOP < 5mmHg) as the commonest complication (*n* = 14, 1.5%), followed by gross hyphema (*n* = 6, 0.6%), and bleb leak (*n* = 4, 0.4%) respectively [7]. Our higher rates of hypotony could be due to a more inclusive definition of hypotony being used (IOP < 6 mmHg, occurring any time postoperatively), the inclusion of patients with intraoperative MMC use only, which has a known association with prolonged hypotony [14], and the longer follow-up period. The incidence of hypotony as a postoperative complication varies greatly between different studies, ranging from as low as 1.5% (Tan et al. 2011) to 38% (Murthy et al. 2006). This is in part due to the non-standardized definitions of hypotony and surgical techniques including the use of different anti-metabolites. In our study, we followed the recommended guidelines for hypotony reporting according to the Guidelines on Design and Reporting of Glaucoma Surgical Trials, stratified by time of onset and severity accordingly. It is interesting to note that though one-quarter of our patients experienced hypotony postoperatively, none required reoperation or experienced significant visual loss of ≥ 2 Snellen lines, suggesting that most transient early postoperative hypotony could be safely managed conservatively if the AC is not flat and hypotony is not prolonged.

To our knowledge, no study has established a correlation between a specific complication and success rates in combined phacotrabeculectomy. Our study has shown that occurrence of early postoperative hypotony was associated with higher risk of drainage failure (IOP > 21 mmHg). Specifically, our study has found that hypotony occurring with other complication(s) simultaneously significantly increased the risk of surgical failure at IOP > 21 mmHg, while both early and late hypotony were associated with higher risk of surgical failure at IOP > 21 mmHg. Benson et al suggested that early hypotony could result in the breakdown of the blood-aqueous barrier, resulting in the release of inflammatory mediators, where persistent conjunctival inflammation could then induce enhanced bleb scarring [15]. In Asian population where the propensity for bleb scarring is greater than in White population, this phenomenon could be amplified and result in poorer surgical outcomes [16]. Prolonged early hypotony could also influence bleb maturation, inhibit the formation of multiform wall reflectivity and microcysts with multiple layers in the crucial first postoperative month. These early bleb morphological features are shown to be predictive of bleb function and surgical success up to six months postoperatively [17]. This could lead to earlier formation of bleb scarring despite adequate MMC application, which may obstruct further outflow of aqueous humor. Hypotony, when accompanied by other complications such as shallow AC and bleb leak, may also hasten the process of bleb scarring and result in higher risk of failure.

In our study, we were unable to identify any risk factors associated with postoperative hypotony. Phacoemulsification has been demonstrated to have a modest sustained reduction in IOP for both patients with POAG and PACG eyes [18, 19, 20]. Previous studies have also reported a rapid and transient decline of IOP after phacotrabeculectomy up to 20% incidence of hypotony postoperatively [21–23]. Concurrent phacoemulsification and trabeculectomy also appear to have an additive effect in IOP reduction, which could predispose patients into postoperative hypotony [24, 25]. Nonetheless, Shingleton et al proposed certain modifications in surgical technique which could reduce postoperative hypotony from 20% to as low as 6.1%, such as 1) using a more posterior keratome entry during phacoemulsification to incorporate the limbal vascular arcade and conjunctiva, 2) using measured phacoemulsification incision length of 2.5 mm instead of estimated length of 2.0 mm, and 3) aiming for a higher case-completion IOP of 20 mmHg [22]. In our study, the majority of eyes with hypotony occurred early postoperatively which subsequently resolved with many achieving IOP in the low teens (mean 13.1 ± 3.8 mmHg at 3 years after surgery). This suggests that the surgeon factor also plays an important role in determining the early post-operative IOP, which includes the tension of sutures, number of sutures, immediate IOP at the end of surgery and timing of suturelysis [26–28].

Interestingly, our study also found that male patients and being non-Chinese were independent risk factors for developing postoperative shallow AC (**Table 3**). In view of the large variance in 95% CI for both readings, however, this study may be inadequately powered for the above assertions. Budenz et al have reported black race as a strong predictor for failure after phacotrabeculectomy [29], though there is no corresponding study which demonstrated any racial or gender predisposition for developing a postoperative complication. More studies would be necessary to evaluate possible racial and gender differences, including the possibility of different cultural and behavioural practices, which may influence aqueous outflow and bleb maturation in the early postoperative period. Notwithstanding, it could be helpful to emphasize the importance of wound care in the postoperative period and remind all patients to avoid applying forceful pressure on the eye.

We recognize the limitations of this study. Being a retrospective study, it was impossible to standardize documentation and the complication rates may have been underreported. For example, we were unable to acquire the full data on corneal endothelial cell count in our patients before and after phacotrabeculectomy, as a reduction of corneal endothelial cell count has been reported and could compromise the safety profile of this operation [30, 31]. Multiple surgeons were in charge of the pre-, intra- and postoperative care of different patients and this could confound documentation errors and introduce systemic biases. We tried to minimize this by conducting a standardized audit with an independent observer who is not employed under NUH department. The low incidence of certain complications also could have introduced greater statistical uncertainty due to random error, or resulted in a lack of statistical power to detect if less frequent complications (e.g. persistent inflammation) were risk factors for failure. Loss to follow-up was another issue as 22% of our patients (*n* = 35) were lost to follow-up within the first three years. We hypothesize that these patients could have defaulted follow-up because they were most likely asymptomatic following the surgery (we noted four patients passed away in the postoperative period). A follow-up duration of three years also appears insufficient to determine the true incidence of blebitis. Longer prospective studies or randomized controlled trials would be preferred to address the above limitations, and at the same time to clearly and better define the advantages of combined phacotrabeculectomy with intraoperative MMC over other therapeutic options for glaucoma in this population.

In conclusion, our study showed that the majority of complications from phacotrabeculectomy with MMC were transient and self-limiting, with hypotony, hyphema and shallow anterior chamber being the most common. Patients could be reassured of the safety of this operation; however, close and active monitoring is critical, especially in the early postoperative period, to prevent the occurrence of prolonged hypotony or multiple complications which may result in surgical failure.

## References

[1] Quigley HA (2011) Glaucoma. Lancet 377: 1367–1377. 10.1016/S0140-6736(10)61423-7 21453963

[2] Cook C, Foster P (2012) Epidemiology of glaucoma: what's new? Can J Ophthalmol 47: 223–226. 10.1016/j.jcjo.2012.02.003 22687296

[3] Foster PJ, Oen FT, Machin D, Ng TP, Devereux JG, Johnson GJ, et al (2000) The prevalence of glaucoma in Chinese residents of Singapore: a cross-sectional population survey of the Tanjong Pagar district. Arch Ophthalmol 118: 1105–1111. 10922206 10.1001/archopht.118.8.1105

[4] Casson RJ, Salmon JF (2001) Combined surgery in the treatment of patients with cataract and primary open-angle glaucoma. J Cataract Refract Surg 27: 1854–1863. 11709261 10.1016/s0886-3350(01)01127-0

[5] AGIS Investigators (2001) The Advanced Glaucoma Intervention Study: 8. Risk of cataract formation after trabeculectomy. Arch Ophthalmol 119: 1771–1779. 11735786 10.1001/archopht.119.12.1771

[6] Husain R, Liang S, Foster PJ, Gazzard G, Bunce C, Chew PT, et al (2012) Cataract surgery after trabeculectomy: the effect on trabeculectomy function. Arch Ophthalmol 130: 165–170. 10.1001/archophthalmol.2011.329 21987579

[7] Tan YL, Tsou PF, Tan GS, Perera SA, Ho CL, Wong TT, et al (2011) Postoperative complications after glaucoma surgery for primary angle-closure glaucoma vs primary open-angle glaucoma. Arch Ophthalmol 129: 987–992. 10.1001/archophthalmol.2011.71 21482857

[8] Belyea DA, Dan JA, Lieberman MF, Stamper RL (1997) Midterm follow-up results of combined phacoemulsification, lens implantation, and mitomycin-C trabeculectomy procedure. J Glaucoma 6: 90–98. 9098816

[9] Law SK, Shih K, Tran DH, Coleman AL, Caprioli J (2009) Long-term outcomes of repeat vs initial trabeculectomy in open-angle glaucoma. Am J Ophthalmol 148: 685–695.e681. 10.1016/j.ajo.2009.05.032 19596220

[10] Sng CC, See JS, Ngo CS, Singh M, Chan YH, Aquino MC, et al (2011) Changes in retinal nerve fibre layer, optic nerve head morphology, and visual field after acute primary angle closure. Eye (Lond) 25: 619–625. 10.1038/eye.2011.31 21436844 PMC3171266

[11] Shaarawy TM, Sherwood MB, Grehn F (2009) Guidelines on Design and Reporting of Glaucoma Surgical Trials. World Glaucoma Association: Kugler Publications. 93 p.

[12] Kirwan JF, Lockwood AJ, Shah P, Macleod A, Broadway DC, King AJ, et al (2013) Trabeculectomy in the 21st century: a multicenter analysis. Ophthalmology 120: 2532–2539. 10.1016/j.ophtha.2013.07.049 24070811

[13] Murthy SK, Damji KF, Pan Y, Hodge WG (2006) Trabeculectomy and phacotrabeculectomy, with mitomycin-C, show similar two-year target IOP outcomes. Can J Ophthalmol 41: 51–59. 16462873 10.1016/S0008-4182(06)80067-0

[14] Kupin TH, Juzych MS, Shin DH, Khatana AK, Olivier MM (1995) Adjunctive mitomycin C in primary trabeculectomy in phakic eyes. Am J Ophthalmol 119: 30–39. 7825687 10.1016/s0002-9394(14)73810-3

[15] Benson S, Mandal K, Bunce C, Fraser S (2005) Is post-trabeculectomy hypotony a risk factor for subsequent failure? A case control study. BMC Ophthalmology 5: 7 15811180 10.1186/1471-2415-5-7 PMC1079872

[16] Wong JS, Yip L, Tan C, Chew P (1998) Trabeculectomy survival with and without intra-operative 5-fluorouracil application in an Asian population. Australian and New Zealand Journal of Ophthalmology 26: 283–288. 9843255 10.1111/j.1442-9071.1998.tb01331.x

[17] Khamar MB, Soni SR, Mehta SV, Srivastava S, Vasavada VA (2014) Morphology of functioning trabeculectomy blebs using anterior segment optical coherence tomography. Indian J Ophthalmol 62: 711–714. 10.4103/0301-4738.136227 25005200 PMC4131325

[18] Shrivastava A, Singh K (2010) The effect of cataract extraction on intraocular pressure. Curr Opin Ophthalmol 21: 118–122. 10.1097/ICU.0b013e3283360ac3 20040874

[19] Hayashi K, Hayashi H, Nakao F, Hayashi F (2001) Effect of cataract surgery on intraocular pressure control in glaucoma patients. J Cataract Refract Surg 27: 1779–1786. 11709251 10.1016/s0886-3350(01)01036-7

[20] Poley BJ, Lindstrom RL, Samuelson TW, Schulze R Jr. (2009) Intraocular pressure reduction after phacoemulsification with intraocular lens implantation in glaucomatous and nonglaucomatous eyes: evaluation of a causal relationship between the natural lens and open-angle glaucoma. J Cataract Refract Surg 35: 1946–1955. 10.1016/j.jcrs.2009.05.061 19878828

[21] Rhee DJ, Deramo VA, Connolly BP, Blecher MH (1999) Intraocular pressure trends after supranormal pressurization to aid closure of sutureless cataract wounds. J Cataract Refract Surg 25: 546–549. 10198861 10.1016/s0886-3350(99)80053-4

[22] Shingleton BJ, Rosenberg RB, Teixeira R, O'Donoghue MW (2007) Evaluation of intraocular pressure in the immediate postoperative period after phacoemulsification. J Cataract Refract Surg 33: 1953–1957. 17964404 10.1016/j.jcrs.2007.06.039

[23] Shingleton BJ, Wadhwani RA, O'Donoghue MW, Baylus S, Hoey H (2001) Evaluation of intraocular pressure in the immediate period after phacoemulsification. J Cataract Refract Surg 27: 524–527. 11311617 10.1016/s0886-3350(00)00641-6

[24] Tham CC, Kwong YY, Leung DY, Lam SW, Li FC, Chiu TY, et al (2008) Phacoemulsification versus combined phacotrabeculectomy in medically controlled chronic angle closure glaucoma with cataract. Ophthalmology 115: 2167–2173.e2162. 10.1016/j.ophtha.2008.06.016 18801576

[25] Chia WL, Goldberg I (1998) Comparison of extracapsular and phaco-emulsification cataract extraction techniques when combined with intra-ocular lens placement and trabeculectomy: short-term results. Aust N Z J Ophthalmol 26: 19–27. 9524026 10.1046/j.1440-1606.1998.00074.x

[26] Ralli M, Nouri-Mahdavi K, Caprioli J (2006) Outcomes of laser suture lysis after initial trabeculectomy with adjunctive mitomycin C. J Glaucoma 15: 60–67. 16378020 10.1097/01.ijg.0000195929.94922.a2

[27] Aykan U, Bilge AH, Akin T, Certel I, Bayer A (2007) Laser suture lysis or releasable sutures after trabeculectomy. J Glaucoma 16: 240–245. 17473738 10.1097/IJG.0b013e31802d6ded

[28] Kapetansky FM (2003) Laser suture lysis after trabeculectomy. J Glaucoma 12: 316–320. 12897576 10.1097/00061198-200308000-00005

[29] Budenz DL, Pyfer M, Singh K, Gordon J, Piltz-Seymour J, Keates EU (1999) Comparison of phacotrabeculectomy with 5-fluorouracil, mitomycin-C, and without antifibrotic agents. Ophthalmic Surg Lasers 30: 367–374. 10334024

[30] Buys YM, Chipman ML, Zack B, Rootman DS, Slomovic AR, Trope GE (2008) Prospective randomized comparison of one-versus two-site Phacotrabeculectomy two-year results. Ophthalmology 115: 1130–1133.e1131. 10.1016/j.ophtha.2007.09.007 18171584

[31] Nassiri N, Nassiri N, Rahnavardi M, Rahmani L (2008) A comparison of corneal endothelial cell changes after 1-site and 2-site phacotrabeculectomy. Cornea 27: 889–894. 10.1097/ICO.0b013e31817618b0 18724149

